# Supplementary material for: Nutritional self-care practices and skills of patients with diabetes mellitus: A study at a tertiary hospital in Ghana
Source: PLoS One. 2022 Mar 23;17(3):e0265608. doi: 10.1371/journal.pone.0265608 (PMC8942245; doi:10.1371/journal.pone.0265608)
Supplement: S1 File — (DOCX) [file pone.0265608.s001.docx]

**S1 file: INTERVIEW GUIDE**

SECTION A: SOCIO-DEMOGRAPHIC INFORMATION

Pseudonym:

1. Age: 18 – 29 ( ), 30 – 39 ( ), 40 – 49 ( ), 50 – 59 ( ), 60 and above ( )
2. Level of education: Primary ( ), JHS ( ), SHS ( ), Tertiary ( ), Nil ( )
3. Gender…………………………………….
4. Duration of diagnosis of condition………………………………
5. Occupation………………………………………
6. Marital status………………………………..
7. Ethnicity/Tribe…………………………….
8. Religion…………………………………..

SECTION B: GUIDING QUESTIONS

1. How do you manage your condition nutritionally?

Probe

1. How many times do you eat in a day?
2. What types of food do you eat during breakfast, lunch and supper?
3. What sources of carbohydrates do you take during breakfast, lunch and supper?
4. What sources of protein do you take do you take during breakfast, lunch and supper?
5. What sources of fats and oil do you take do you take during breakfast, lunch and supper?
6. What is the effect of the types of food you eat on your blood glucose level?
7. What is the quantity of food you usually eat during breakfast, lunch and supper?

Probe

1. What is the effect of the quantity of food you eat during breakfast, lunch and supper on your blood glucose level?
2. How do you regulate your carbohydrates intake and eating pattern?

Probe

1. How often do you read and make use of food labels when buying food products?
2. What are the things you take into consideration on food labels before you buy or consume food products with labels?
3. How do you regulate your intake of soft drinks?
4. How do you regulate your intake of alcoholic drinks?
5. How do you plan your meals as a means of managing your condition nutritionally?

Probe

1. What are the things or factors you consider when planning your meals?
2. Is there anything else you will like to tell me?
