# Supplementary material for: Nutritional self-care practices and skills of patients with diabetes mellitus: A study at a tertiary hospital in Ghana
Source: PLoS One. 2022 Mar 23;17(3):e0265608. doi: 10.1371/journal.pone.0265608 (PMC8942245; doi:10.1371/journal.pone.0265608)
Supplement: Attachment — Submitted filename: Response to reviewers.docx [file pone.0265608.s002.docx]

**Response to Review Decision	29/12/2021**

**Manuscript number: PONE-D-21-20315**

**Title of the manuscript: Nutritional self-care practices and skills of patients with diabetes mellitus: A study at a tertiary hospital in Ghana**

Dear Editor,

We are so grateful for giving us the opportunity to revise our manuscript for resubmission. The suggestions from the reviewers and editor have greatly improved the manuscript. All the concerns raised by the reviewers have been addressed accordingly. We thank you and the reviewers for your comments.

Below are our responses to the points raised by you and the reviewers.

All authors have agreed to the revisions made in the manuscript for submission.

Yours sincerely, a

Gwendolyn Patience Mensah (PhD)

(corresponding author)

**EDITOR:**

**1. We suggest you thoroughly copyedit your manuscript for language usage, spelling, and grammar.**

The manuscript has been edited by an academic writer who does professional scientific editing. All the grammatical errors have been corrected by the academic writer who edited the manuscript.

She is Ms. Vicki Igglesden, Nelson Mandela University, Port Elizabeth, South Africa.

**2. When reporting the results of qualitative research, we suggest consulting the COREQ guidelines: http://intqhc.oxfordjournals.org/content/19/6/349. In this case, please consider including more information on the number of interviewers, their training and characteristics; and please provide the interview guide used.**

On lines 10 to 12 of page 6, information on who conducted the interviews has been clearly stated. The interview guide for the study has also been provided as an attachment to the manuscript.

**REVIEWER #1:**

**1. Is the manuscript presented in an intelligible fashion and written in standard English?**
**PLOS ONE does not copyedit accepted manuscripts, so the language in submitted articles must be clear, correct, and unambiguous. Any typographical or grammatical errors should be corrected at revision, so please note any specific errors here.**

**Reviewer #1: No**

The manuscript has been edited by an academic writer who does professional scientific editing. The typographical and grammatical errors have been corrected by the academic writer who edited the manuscript.

**2. Most of the key words are not MeSH terms.**

The keywords that were not MeSH terms have now been changed to MeSH terms. This can be found on the last line of page 2.

**3. Scientific or otherwise basis of choosing 15 participants not explained.**

The scientific rationale or basis for choosing 15 participants has now been clearly stated on lines 15 to 18 of page 5.

**4. Results section of abstract does not show any numerical or percentage value.**

The results section of the abstract has now been edited to include numerical values. This can be found on page 2.

**5. Literature review on subject is not up to the mark.**

Literature has been reviewed on the subject. On lines 15 to 18 of page 3 and lines 7 to 11 of page 4, new literature has been added to the manuscript.

**6. Sampling technique is not mentioned.**

The sampling technique that was used for the study has been clearly stated on line 11 of page 5.

**7. Values of findings in results should be mentioned as percentage points or qualifiers like half of participants, one third, one fourth etc. Absolute numbers are avoided in qualitative studies.**

This has been corrected on all the pages of the results section of the manuscript.

**8. Grammar needs to be improved.**

The manuscript has been edited by an academic writer who does professional scientific editing. All the grammatical errors have been corrected by the academic writer who edited the manuscript.

**REVIEWER #2:**

**1. Is the manuscript technically sound, and do the data support the conclusions?**

**The manuscript must describe a technically sound piece of scientific research with data that supports the conclusions. Experiments must have been conducted rigorously, with appropriate controls, replication, and sample sizes. The conclusions must be drawn appropriately based on the data presented.**

**Reviewer #2: No**

The manuscript has been edited by an academic writer who does professional scientific editing. All the grammatical and technical errors have been corrected by the academic writer who edited the manuscript. The scientific basis for the sample size used has been stated on lines 15 to 18 of page 5. We believe the corrections effected have made the manuscript technically sound.

**2. Have the authors made all data underlying the findings in their manuscript fully available?
The PLOS Data policy requires authors to make all data underlying the findings described in their manuscript fully available without restriction, with rare exception (please refer to the Data Availability Statement in the manuscript PDF file). The data should be provided as part of the manuscript or its supporting information, or deposited to a public repository. For example, in addition to summary statistics, the data points behind means, medians and variance measures should be available. If there are restrictions on publicly sharing data—e.g. participant privacy or use of data from a third party—those must be specified.**

**Reviewer #2: No**

We believe that the data underlying the findings of the study has been indicated or provided in the manuscript. The results section of the manuscript contains numerous direct quotes from the participants of the study.

**3. Is the manuscript presented in an intelligible fashion and written in standard English?

PLOS ONE does not copyedit accepted manuscripts, so the language in submitted articles must be clear, correct, and unambiguous. Any typographical or grammatical errors should be corrected at revision, so please note any specific errors here.**

**Reviewer #2: No**

The manuscript has been edited by an academic writer who does professional scientific editing. The typographical and grammatical errors have all been corrected by the academic writer who edited the manuscript.

**4. The present study has many limitations, majorly sample size is very less and due to this it can’t provide definite conclusions.**

The scientific rationale for the sample size used has been indicated on lines 15 to 18 of page 5. The sample size was determined by saturation. We believe this justifies the conclusions drawn.

**5. Inclusion: authors considered both type 1 and type 2 diabetes.**

This has clearly been indicated on lines 12 and 13 of page 5.
